# Molecular Chaperone Mediated Late-Stage Neuroprotection in the SOD1^G93A^ Mouse Model of Amyotrophic Lateral Sclerosis

**DOI:** 10.1371/journal.pone.0073944

**Published:** 2013-08-30

**Authors:** Sergey S. Novoselov, Wendy J. Mustill, Anna L. Gray, James R. Dick, Naheed Kanuga, Bernadett Kalmar, Linda Greensmith, Michael E. Cheetham

**Affiliations:** 1 UCL Institute of Ophthalmology, London, United Kingdom; 2 Sobell Department of Motor Neuroscience and Movement Disorders, UCL Institute of Neurology, London, United Kingdom; 3 MRC Centre for Neuromuscular Diseases, UCL Institute of Neurology, London, United Kingdom; UMCG, The Netherlands

## Abstract

Amyotrophic lateral sclerosis (ALS) is a fatal neurodegenerative disorder characterized by the selective loss of motor neurons in the spinal cord, brain stem, and motor cortex. Mutations in superoxide dismutase (SOD1) are associated with familial ALS and lead to SOD1 protein misfolding and aggregation. Here we show that the molecular chaperone, HSJ1 (DNAJB2), mutations in which cause distal hereditary motor neuropathy, can reduce mutant SOD1 aggregation and improve motor neuron survival in mutant SOD1 models of ALS. Overexpression of human HSJ1a (hHSJ1a) *in vivo* in motor neurons of SOD1^G93A^ transgenic mice ameliorated disease. In particular, there was a significant improvement in muscle force, increased motor unit number and enhanced motor neuron survival. hHSJ1a was present in a complex with SOD1^G93A^ and led to reduced SOD1 aggregation at late stages of disease progression. We also observed altered ubiquitin immunoreactivity in the double transgenic animals, suggesting that ubiquitin modification might be important for the observed improvements. In a cell model of SOD1^G93A^ aggregation, HSJ1a preferentially bound to mutant SOD1, enhanced SOD1 ubiquitylation and reduced SOD1 aggregation in a J-domain and ubiquitin interaction motif (UIM) dependent manner. Collectively, the data suggest that HSJ1a acts on mutant SOD1 through a combination of chaperone, co-chaperone and pro-ubiquitylation activity. These results show that targeting SOD1 protein misfolding and aggregation *in vivo* can be neuroprotective and suggest that manipulation of DnaJ molecular chaperones might be useful in the treatment of ALS.

## Introduction

Amyotrophic lateral sclerosis (ALS) is a progressive, adult-onset neurodegenerative disorder characterized by the selective loss of upper and lower motor neurons in the brain and spinal cord. The loss of motor neurons results in progressive paralysis and death, typically within 5 years of onset [1]. Most cases of ALS are sporadic and of unknown cause (sporadic ALS, or sALS), however, in around 10% of cases the disease is inherited (familial ALS, or fALS). Of these, approximately 20% have been attributed to mutations in the gene encoding superoxide dismutase 1 (SOD1) [2]. Several potential disease mechanisms have been proposed for mutant SOD1 mediated motor neuron cell death [3], [4]; however, the critical initiators and accelerators of motor neuron degeneration remain elusive.

A hallmark of many neurodegenerative diseases, including ALS, is the formation of ubiquitin positive inclusions of aggregated protein. Intracellular proteinaceous inclusions, evident in the motor neurons of ALS patients [5], [6], [7] and also in mutant SOD1 mice [8], [9], represent an important feature of ALS. The accumulation of misfolded and aggregation-prone protein suggests an imbalance of protein homeostasis (proteostasis). Molecular chaperones are critical factors for maintaining proteostasis through facilitating protein folding and quality control [10]. Indeed, the heat shock response co-inducer arimoclomol, which upregulates molecular chaperone expression, can protect against mutant SOD1 toxicity *in vivo* [11], [12]. The manipulation of several individual molecular chaperones and co-chaperones, including small Hsps (HSPB1, HSPB5, HSPB8), Hsp70 (HSPA1), DNAJB1, CHIP and BAG1 have all been shown to reduce mutant SOD1 aggregation and/or improve viability in cells [13], [14], [15], [16], [17], [18], [19]. However, the upregulation of individual chaperones *in vivo* has been less successful, as only HSPB1 provided some protective effects at an early phase in the mutant SOD1^G93A^ model of ALS, but not at late stage of the disease [17], [20]. Overexpression of either HSPA1 or BAG1 had no beneficial effects in mouse models of ALS [15], [18].

The molecular chaperone HSJ1 (DNAJB2) is a member of the Hsp40 (or DnaJ) family of heat shock proteins that contain a J domain, which is crucial in substrate recognition by Hsp70 [21], [22], [23]. Thus, Hsp40/DnaJ protein function is essential for Hsp70 function, including protein folding and directing misfolded proteins towards the proteasome [23]. HSJ1 is preferentially expressed in neurons as two alternatively spliced isoforms that differ in their subcellular localisation [21], [24]. The smaller cytoplasmic isoform, HSJ1a (DNAJB2a), can suppress the aggregation of polyglutamine expanded proteins [25], [26], [27], [28], through a mechanism dependent on the regulation of the Hsp70 ATPase cycle by its J domain and enhancing proteasomal degradation of misfolded client proteins via its ubiquitin interaction motif (UIM) domains [25]. HSJ1a can also co-operate with Hsp70 to regulate the proteasomal targeting of the spinocerebellar ataxia type 3-linked protein, ataxin 3 [29]. Transgenic upregulation of HSJ1a in a mouse model of Huntington's disease reduced huntingtin aggregation and improved neurological performance by reducing the ability of aggregated huntingtin to promote further aggregation [28]. Furthermore, HSJ1 appears to be critical for normal motor neuron function, as mutations in *DNAJB2* cause recessive distal hereditary motor neuropathy (dHMN) [30]. The potential importance of HSJ1 for motor neuron function was further demonstrated in a cell model of ALS, in which overexpression of HSJ1a or HSJ1b reduced inclusion formation by the A4V mutant of SOD1 [30].

Therefore, it is possible that enhanced expression of HSJ1a alone might be beneficial in mutant SOD1 induced ALS and could modify SOD1 proteostasis and/or enhance motor neuron function in ALS. In this study, we investigated the ability of HSJ1a to protect against mutant SOD1^G93A^ *in vivo* and in cells. Using a transgenic approach, we expressed human HSJ1a in mice that overexpress the SOD1^G93A^ mutation and assessed physiological function at a late stage of disease to test whether upregulation of HSJ1a can have beneficial effects in this model of ALS. We also assessed the effects of HSJ1a upregulation on spinal cord motor neuron survival and identified interactions between mutant SOD1 protein and HSJ1a in both spinal cords of transgenic mice and in a cell model. Our results show that HSJ1a reduces mutant SOD1 aggregation dependent on its J and UIM domains and can enhance late stage motor neuron survival in a mouse model of SOD1 ALS.

## Results

### hHSJ1a expression in SOD1^G93A^ mice

**Figure 1:**
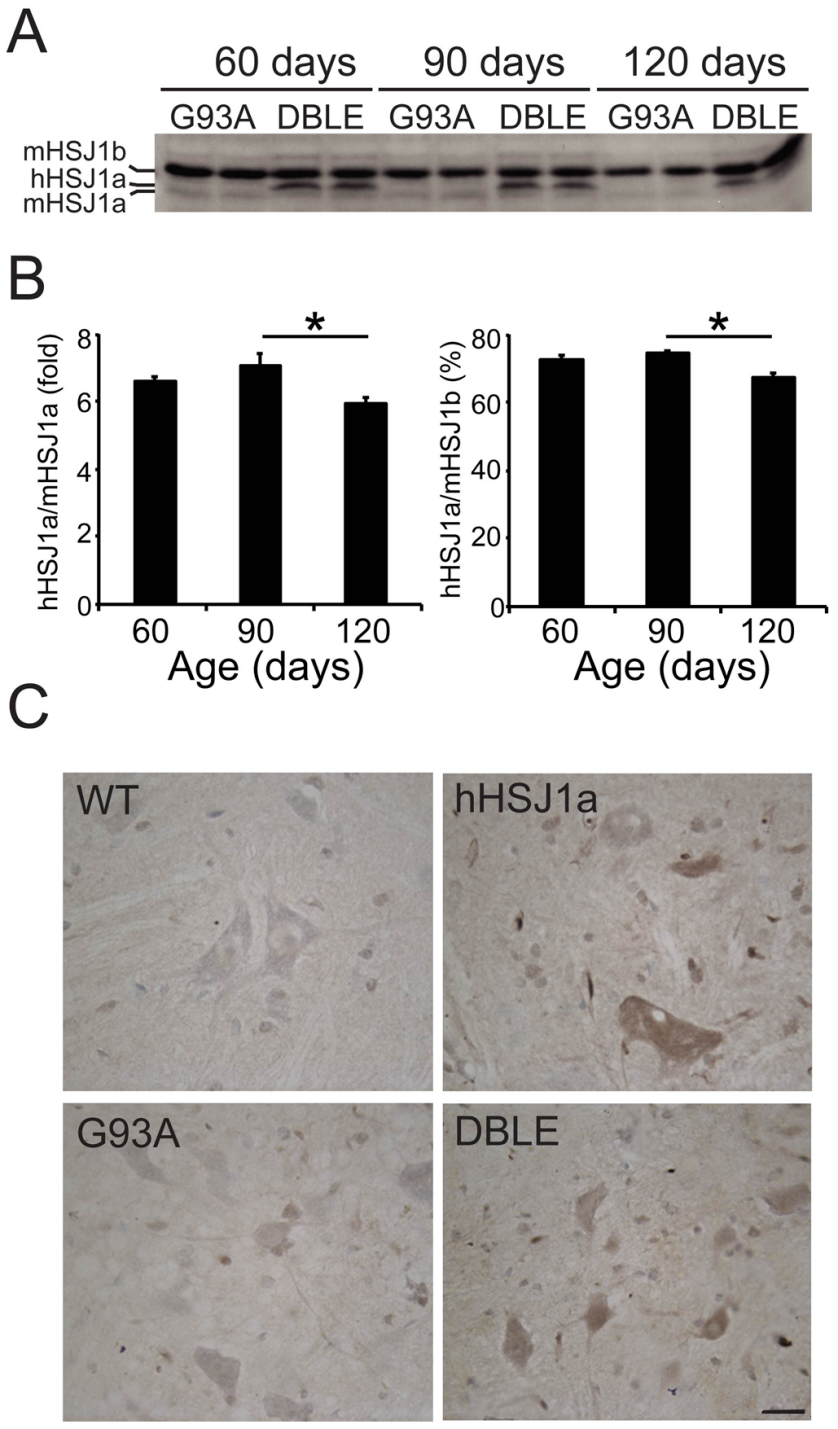
hHSJ1a expression in spinal cord. (**A**) Representative Western blot showing total levels of HSJ1 expression in G93A and DBLE transgenic animals at 60, 90 and 120 days of age using anti-HSJ1 antibody (S653). Note the human HSJ1a (hHSJ1a) transgene band running just above endogenous mouse HSJ1a (mHSJ1a) and below mouse HSJ1b (mHSJ1b). (**B**) hHSJ1a levels in spinal cords were analysed by densitometry and normalised to mHSJ1a (left panel) or mHSJ1b (right panel). Mean relative expression was plotted ±SEM for each age group (n≥4; *p<0.05). (**C**) Representative images of anti-HSJ1a (16321) immunohistochemistry in thoracic region of spinal cords of WT, hHSJ1a, G93A and DBLE transgenic mice at 120 days of age confirming hHSJ1a expression in motor neurons. Scale bar: 20 µm.

HSJ1a transgenic mice were produced that express human HSJ1a (hHSJ1a) under the control of the bovine prion protein promoter. Female hemizygous hHSJ1a and male hemizygous SOD1^G93A^ (G93A) mice [31] were then crossed to generate double transgenic (DBLE) mice. These DBLE mice were compared with littermate G93A, wild type (WT) and hemizygous hHSJ1a only (hHSJ1a) animals. hHSJ1a expression in the spinal cord was assessed by Western blotting and immunohistochemistry (Figure 1A and 1C). The expression of hHSJ1a was approximately seven-fold the endogenous mouse HSJ1a and was relatively constant at 60 and 90 days, but was reduced at 120 days of age (Figure 1B). The constant expression in this line, line 61a, is in contrast to another hHSJ1a transgenic line, line 52a, which showed a three-fold increase in expression between 30 and 105 days of age [28]. Immunohistochemistry of 120 day lumbar spinal cord confirmed that the increase in HSJ1a staining in the hHSJ1a and DBLE animals was predominantly originating from motor neurons (Figure 1C). This suggests that the reduction in hHSJ1a expression in the DBLE spinal cords at 120 days is likely to reflect a reduction in the number of surviving motor neurons due to SOD1^G93A^ toxicity, but might also reflect a reduction in promoter activity or hHSJ1a stability.

### hHSJ1a overexpression does not alter the decline in body weight during disease progression in SOD1^G93A^ mice

**Figure 2:**
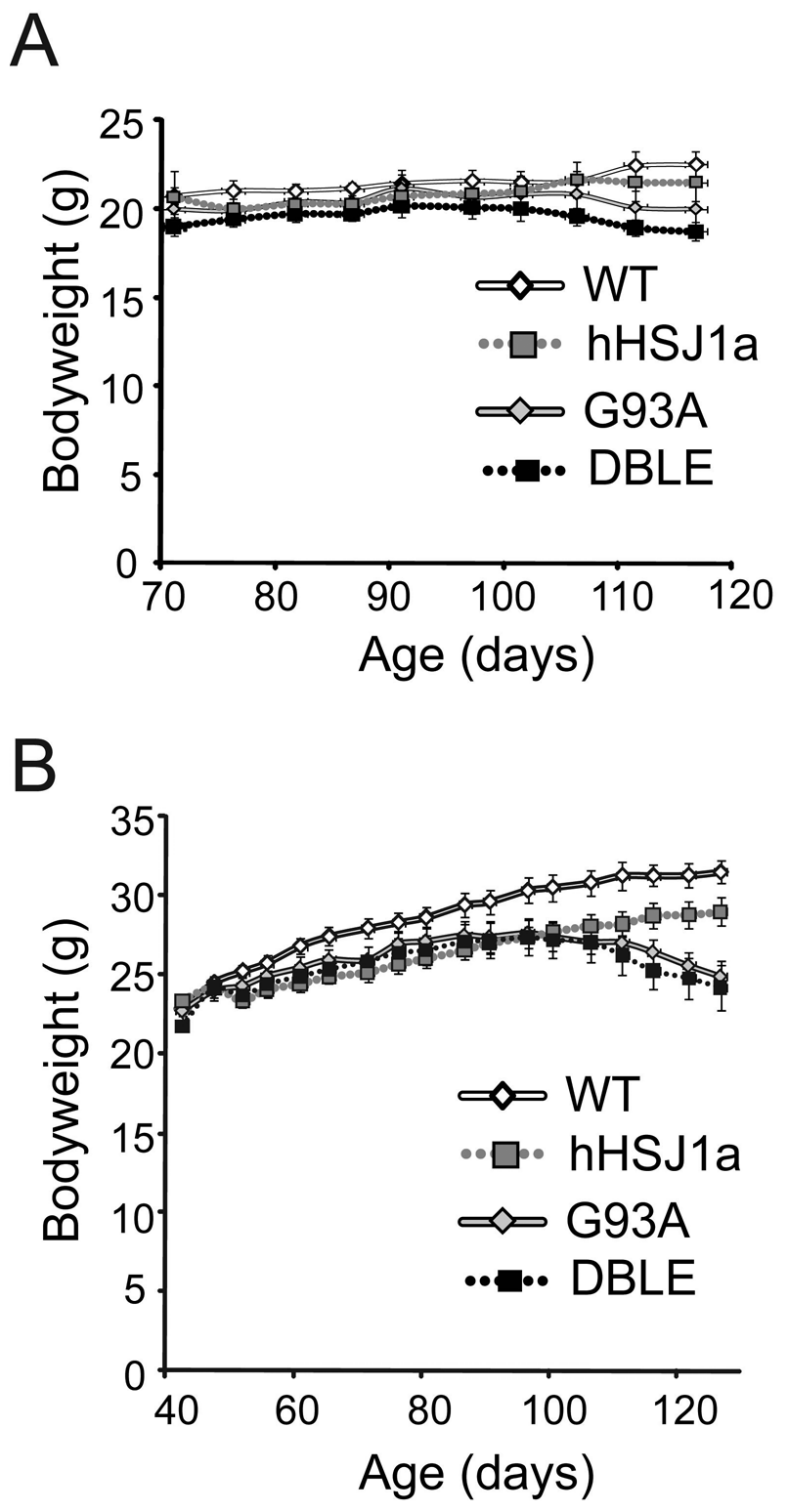
hHSJ1a does not affect the bodyweight of SOD1^G93A^ mice. (**A**) Female and (**B**) male WT, (white line) hHSJ1a (grey dotted), G93A (grey line) or DBLE (black dotted) mice were weighed at least twice weekly from 70–120 days of age. The mean bodyweight ±SEM is shown (n≥7).

Littermate mice from each experimental group (WT, hHSJ1a, G93A and DBLE) were monitored for loss of body weight throughout the study. As previously reported [32], G93A mice started to lose weight from 90 days compared to WT and hHSJ1a mice. Weight loss in female (Figure 2A) and male (Figure 2B) DBLE transgenic mice expressing SOD1^G93A^ and hHSJ1a was very similar to G93A littermates and no significant effect on the loss of bodyweight was observed.

### hHSJ1a overexpression improves muscle function in late stage SOD1^G93A^ mice

**Figure 3:**
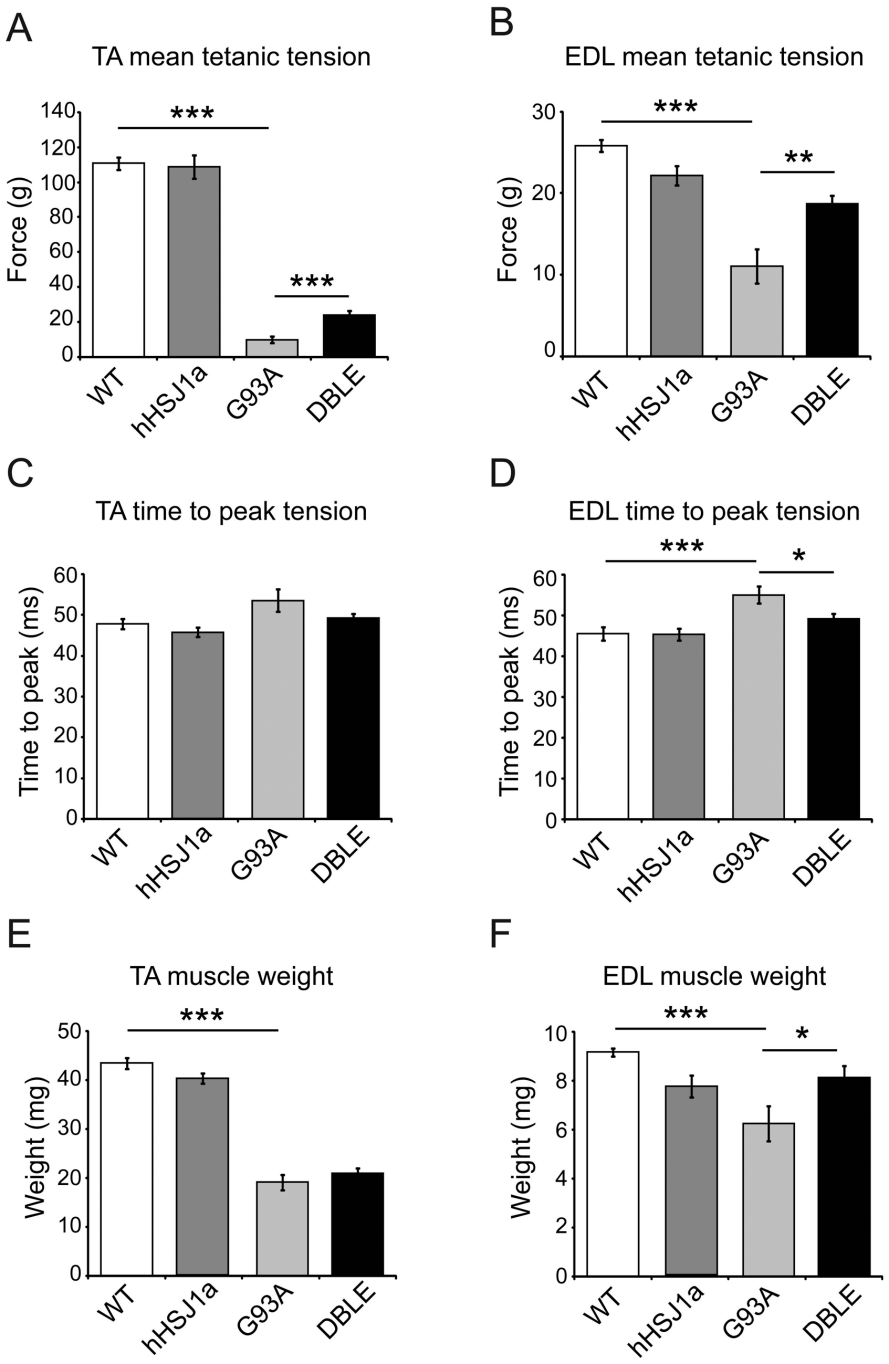
hHSJ1a expression improves muscle characteristics in SOD1^G93A^ mice. Female mice were assessed at 120±5 days. (**A**) Maximal tetanic force generated by the tibialis anterior (TA) muscle (n≥8). (**B**) Maximal tetanic force generated by the extensor digitorum longus (EDL) muscle (n≥10). (**C**) Time to peak (TTP) force for the TA muscle was increased in G93A animals, and in (**D**) EDL this increase in TTP was partially reduced in DBLE (n≥10). (**E**) Mean weight of the TA and (**F**) EDL muscles (n = 12). All graphs show the mean ±SEM, (***p<0.001**p<0.01, *p<0.05).

At 120 days of age, female mice of all genotypes were anaesthetized and underwent *in vivo* assessment of neuromuscular function, including i) assessment of maximum force production in the tibialis anterior (TA) and extensor digitorum longus (EDL) muscles, ii) evaluation of muscle contractile characteristics and iii) assessment of the number of surviving functional motor units innervating the EDL muscle. Similar to previous reports [11], [33], by 120 days the TA and EDL muscles in G93A mice produced approximately 90% and 60% less force than that produced by TA and EDL muscles respectively in WT mice (Figure 3A, B). However, in SOD1^G93A^ mice overexpressing HSJ1a there was a significant preservation of muscle force production, such that TA muscles of DBLE mice were capable of producing 23.9±2.4 g force, compared to 9.9±1.8 g produced by G93A TA muscles (p<0.001; Figure 3A). In EDL muscles there was also an improvement in muscle force production in DBLE mice as these mice were capable of exerting 18.7±1.1 g force, compared to 11.0±2.1 g in G93A mice (p<0.01; Figure 3B).

TA and EDL are fast twitch muscles that normally contract and relax rapidly. However, as a result of the selective loss of motor neurons that innervate fast twitch type IIa muscles fibres in G93A mice as disease progresses, the contractile characteristics of TA and EDL alter, and these fast twitch muscles begin to resemble slow twitch muscles, with a decrease in the speed of muscle contraction [11], [32], [33]. Thus, in G93A mice, the time taken by both TA and EDL to reach maximum twitch force (time to peak; TTP) was significantly slower than in WT mice (Figure 3C, D). In contrast, in EDL muscles of DBLE mice, the TTP was significantly faster than in G93A EDL muscles and was similar to that in WT mice (TTP in G93A EDL muscles = 55.0±2.0 msec; TTP in DBLE = 49.1±1.3 msec compared to TTP in WT = 45.4±1.6 msec, p<0.05 Figure 3D). Although, the TTP of TA in DBLE mice was also faster than in G93A, this improvement did not reach statistical significance (Figure 3C).

### Overexpression of hHSJ1a improves the survival of functional motor units in SOD1^G93A^ mice

**Figure 4:**
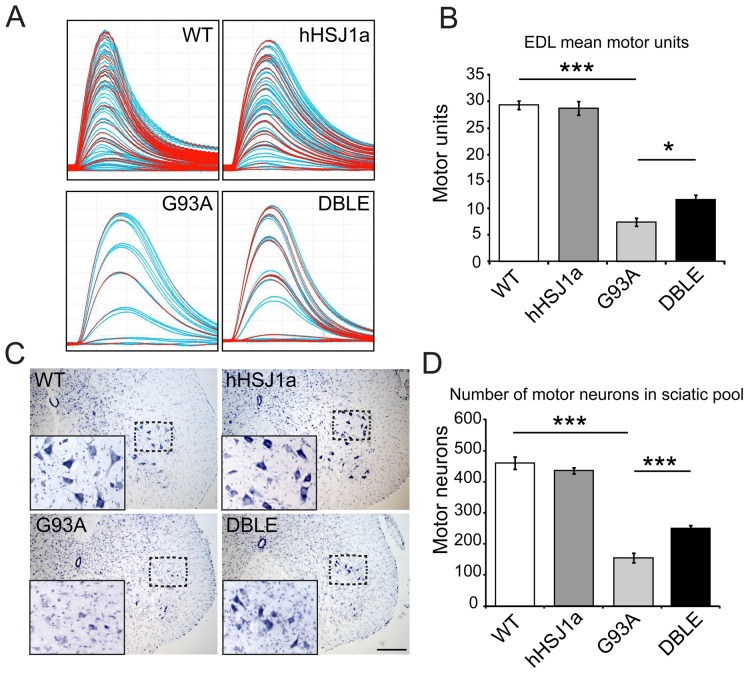
hHSJ1a expression improves motor unit and motor neuron survival in SOD1^G93A^ mice. (**A**) Typical examples of motor unit traces are shown from the EDL muscle of WT, hHSJ1a, G93A and DBLE female mice. (**B**) The mean motor units in the EDL muscle ±SEM are shown in the bar chart (n≥10; ***p<0.001, *p<0.05). (**C**) Typical histological staining of a lumbar spinal cord section of 120 day WT, hHSJ1a, G93A and DBLE female mice. Sections were stained with gallocynanin. Inset shows magnification of boxed area. Scale bar 200 µm. (**D**) Serial gallocynanin stained sections of the L2–L6 region of 120 day spinal cord were analysed under light microscopy and motor neurons counted. The bar chart shows the mean motor neuron number ±SEM (n = 3; ***p<0.001, **p<0.01).

The number of functional motor units innervating EDL muscles was then assessed *in vivo* in mice of each experimental group. Typical motor unit recordings for each experimental group are shown in Figure 4A. Assessment of the number of motor units revealed that overexpression of hHSJ1a alone had no effect on motor unit number compared to WT (Figure 4B). However, in G93A mice by 120 days of age there is a significant reduction in the number of surviving motor units innervating the EDL, such that only 7.0±0.8 motor units survive in G93A mice, compared to 29.0±1.3 in EDL of WT mice (p<0.001 4B). Importantly, in DBLE mice there was a 57% increase in EDL motor unit survival compared to the G93A (DBLE = 11.0±1.1; p<0.05). These results show that increased hHSJ1a expression protects functional motor units *in vivo* from mutant SOD1^G93A^ toxicity.

### Overexpression of hHSJ1a improves muscle weight and increases motor neuron survival, but has no effect on lifespan of SOD1^G93A^ mice

At the end of the physiological assessment of muscle force, the TA and EDL muscles were removed and weighed in order to determine the extent of muscle mass. As expected, in G93A mice there was a significant reduction in the weight of the TA and EDL muscles compared to WT mice (Figure 3E, F). In DBLE mice, TA muscle weights mice were not significantly greater than in G93A mice (Figure 3E); however, the EDL muscles from DBLE animals weighed significantly more than in mice expressing SOD1^G93A^ alone (Figure 3F; 6.3±0.7 mg in G93A and 8.1±0.5 mg in DBLE EDL; p<0.05).

Following removal of the hindlimb muscles, the animals were perfused with fixative and the lumbar spinal cord removed and processed for determination of motor neuron survival. Motor neurons within the sciatic motor pool were identified in the ventral horn of the lumbar cord (between the lumbar regions L2–L6) using morphological criteria (examples shown in Figure 4C). As can be seen in Figure 4D, and in line with previous reports [11], [33], the number of motor neurons in the spinal cord of G93A mice was dramatically less than in either WT or hHSJ1a mice (WT = 460±20; hHSJ1a = 435±10 compared to 155±15 in G93A; p<0.001 Figure 4D). However, in DBLE transgenic mice expressing hHSJ1a and SOD1^G93A^, there was a 61% increase in motor neuron survival compared to G93A mice (250±10; p<0.001). These morphological data reflect those of motor unit survival (Figure 4B), and confirm that hHSJ1a overexpression protects motor neurons against mutant SOD1^G93A^ toxicity at 120 days of age.

**Figure 5:**
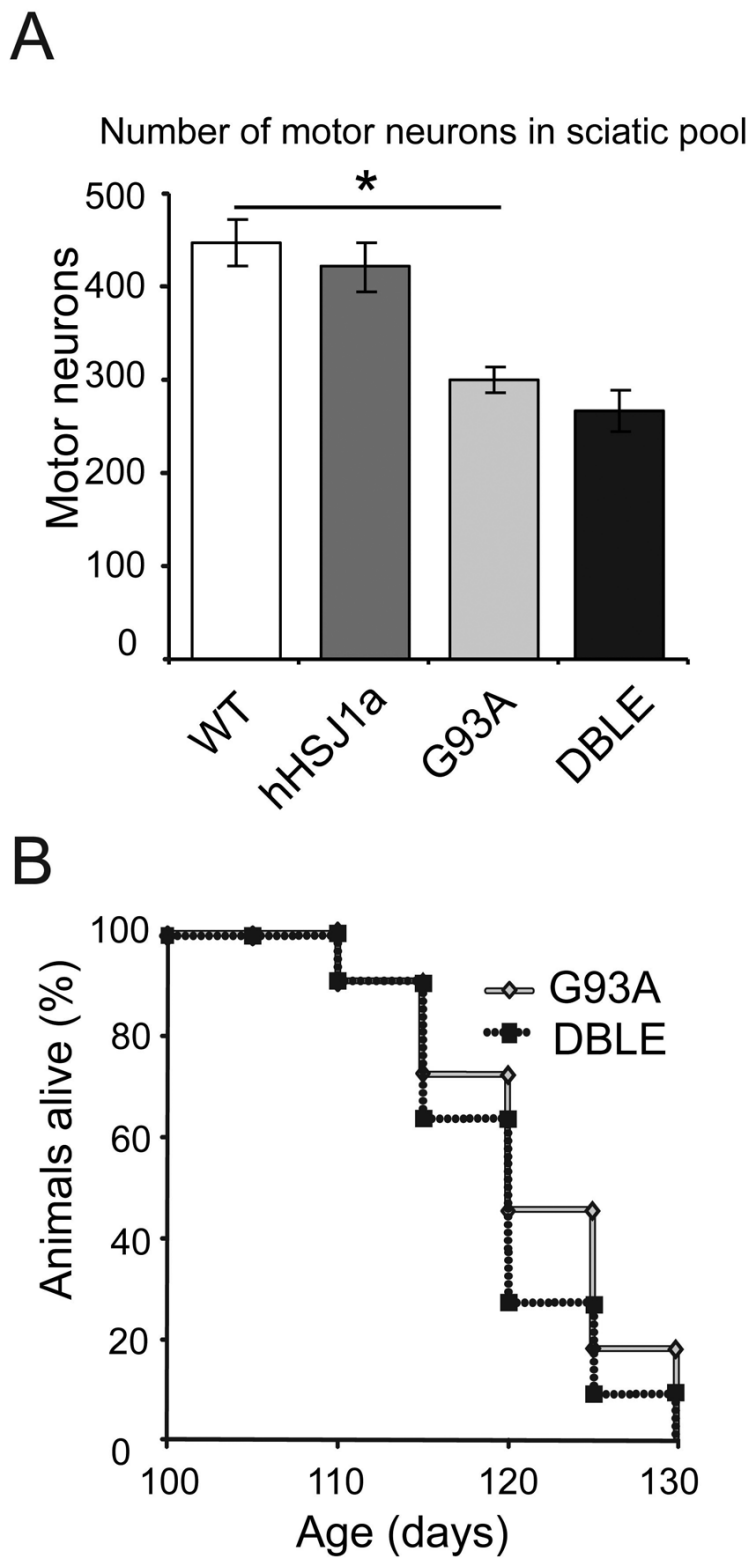
hHSJ1a expression does not improve motor neuron survival at 90 days or lifespan of SOD1^G93A^ mice. (**A**) Serial gallocynanin stained sections of the L2–L6 region from 90 day old spinal cord from female mice were analysed under light microscopy and motor neurons counted. The bar chart shows the mean motor neuron number ±SEM (n = 3; *p<0.05). (**B**) Mean survival of male G93A and DBLE mice is shown (n≥11). Overexpression of hHSJ1a had no effect on the lifespan of SOD1^G93A^ mice.

Given these improvements in motor neuron survival and muscle function at a relatively late stage of disease, we hypothesised that the effect of HSJ1a expression may be more pronounced earlier in disease. To test this hypothesis, motor neurons within the sciatic motor pool of female mice were counted at 90 days of age. The G93A mice had significantly fewer motor neurons compared to WT (67% of WT level, p<0.05; Figure 5A). Surprisingly, the DBLE mice had a similar reduction in motor neurons suggesting that hHSJ1a expression does not improve motor neuron survival at 90 days, and the beneficial effects are only evident late in disease. Furthermore, analysis of lifespan of male G93A and DBLE mice revealed that overexpression of HSJ1a had no significant effect on the lifespan of SOD1^G93A^ mice (Figure 5B).

### hHSJ1a forms a complex with SOD1 and reduces SOD1 aggregation in vivo

**Figure 6:**
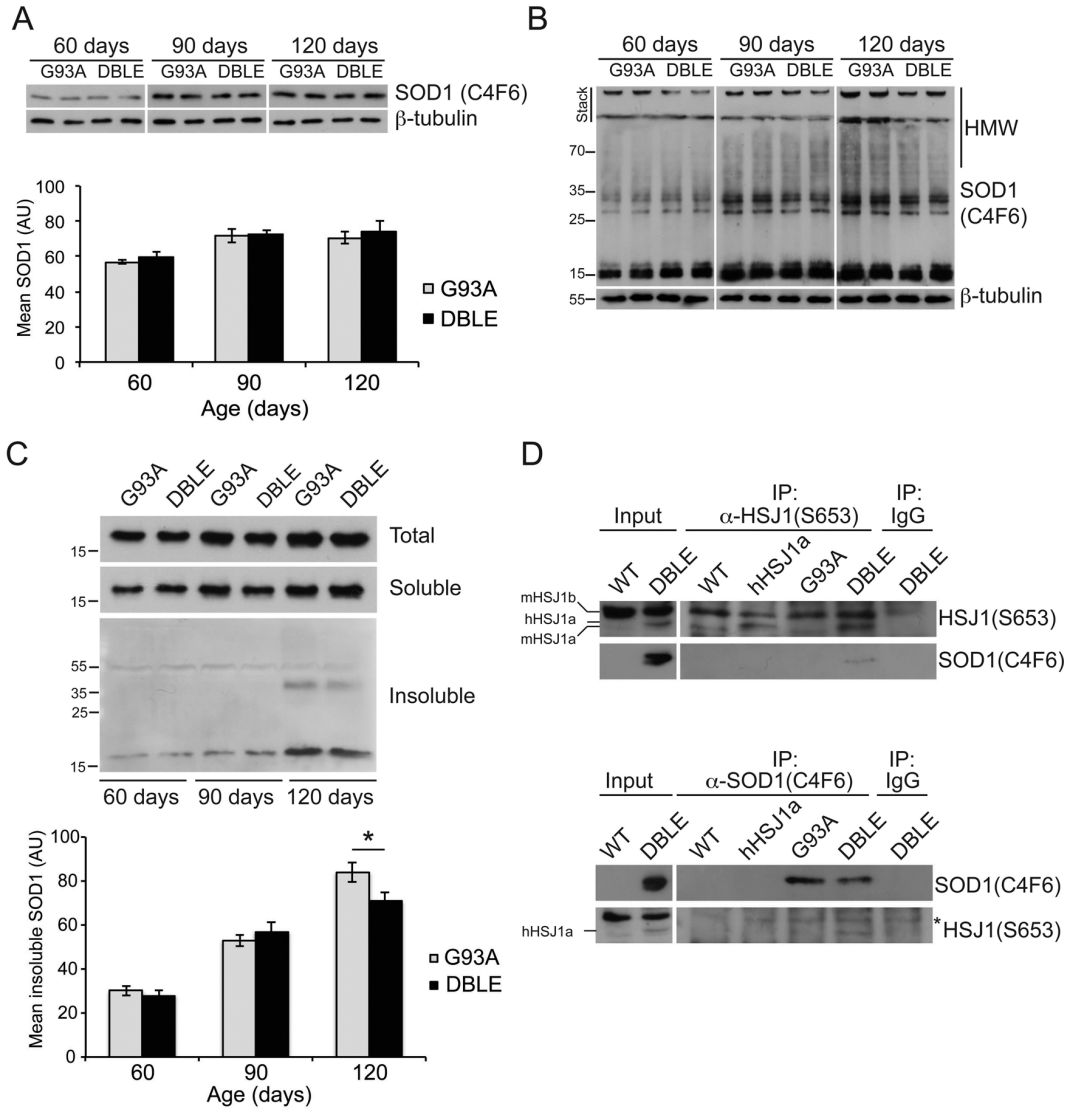
hHSJ1a reduces levels of aggregated SOD1 in spinal cord. (**A**) Representative Western blot with anti-SOD1 (C4F6) of spinal cord lysates from male transgenic animals of indicated age resolved under reducing conditions. Mutant SOD1 levels were quantified by densitometry, expressed relative to β-tubulin and plotted as mean ±SEM for G93A (light grey) and DBLE (black) bars data from at least four independent spinal cords per group. (**B**) Representative Western blots of spinal cord lysates from indicated ages of male mice with anti-SOD1 (C4F6) resolved by SDS-PAGE in non-reducing conditions. Note the decrease in higher molecular weight (HMW) oligomers of SOD1 in DBLE spinal cords. The position of molecular weight markers in indicated on the left in kDa (**C**) Representative Western blots of total, soluble and insoluble SOD1 following differential sedimentation resolved by reducing SDS-PAGE from G93A and DBLE transgenic male animals at the ages indicated stained with anti-SOD1 (C4F6) confirmed the age dependent increase in detergent insoluble SOD1 which was reduced by overexpression of hHSJ1a. Levels of pelleted detergent-insoluble SOD1 were measured by densitometry based on at least four independent spinal cords and plotted as mean ±SEM for G93A (light grey) and DBLE (black) bars for each age group (n≥4; *p<0.05). (**D**) hHSJ1a associates with SOD1 in spinal cords of DBLE transgenic male animals. Western blots of reciprocally immunoprecipitated material (IP) probed for HSJ1 and SOD1. IPs were performed from WT, hHSJ1a, G93A and DBLE transgenic spinal cord lysates at 120 days of age with anti-HSJ1 (S653), SOD1 (C4F6) or control antibodies (IgG), as indicated. Asterisk denotes non-specific band that is characteristic for the antibody combination used.

As overexpression of hHSJ1a protected spinal cord motor neurons and improved muscle function *in vivo* in a model of SOD1^G93A^ ALS, we hypothesised that the protective effect of hHSJ1a in motor neurons might be a result of reducing SOD1 aggregation. The levels of SOD1 protein in the spinal cord of male mice were analysed using the C4F6 antibody that recognizes a misfolded conformation of the SOD1 protein [34], [35]. Analyses of total spinal cord lysates from each genotype confirmed that levels of SOD1 increased as disease progressed in both the G93A and DBLE animals, and hHSJ1a expression did not alter the total level of SOD1 at any stage (Figure 6A). We then used non-reducing SDS-PAGE to monitor the formation of SOD1 aggregates and observed that higher molecular weight (HMW) species that were trapped at the top of the gel, in the stacking gel and in the loading well accumulated as disease progressed from 60 to 120 days. Interestingly, in the DBLE spinal cords there appeared to be less HMW material trapped at the stacking gel and in the well (Figure 6B). To analyse these potential aggregates further, a differential sedimentation protocol of spinal cord lysates was developed to separate soluble protein from sedimentable, aggregated, insoluble protein. Analysing these fractions by Western blotting with C4F6 confirmed that insoluble, aggregated SOD1 accumulated as disease progressed. Importantly, the amount of insoluble SOD1 was significantly reduced in the DBLE spinal cord, but only at 120 days of age, not at earlier stages of disease (Figure 6C). To test the hypothesis that HSJ1a could be acting directly on SOD1, co-immunoprecipitation with C4F6 was used to detect potential HSJ1a:SOD1 complexes. hHSJ1a only precipitated with SOD1 from the DBLE tissue and the reciprocal precipitation confirmed that SOD1 could only be recovered with HSJ1a from the DBLE spinal cords (Figure 6D). Interestingly, mHSJ1a and mHSJ1b were not detected in the C4F6 precipitation only hHSJ1a, despite the higher levels of mHSJ1b in the cell lysates. This suggests that either HSJ1a preferentially binds mutant SOD1, or that the heterologously expressed hHSJ1a protein is more available to bind mutant SOD1 than endogenous HSJ1 proteins.

**Figure 7:**
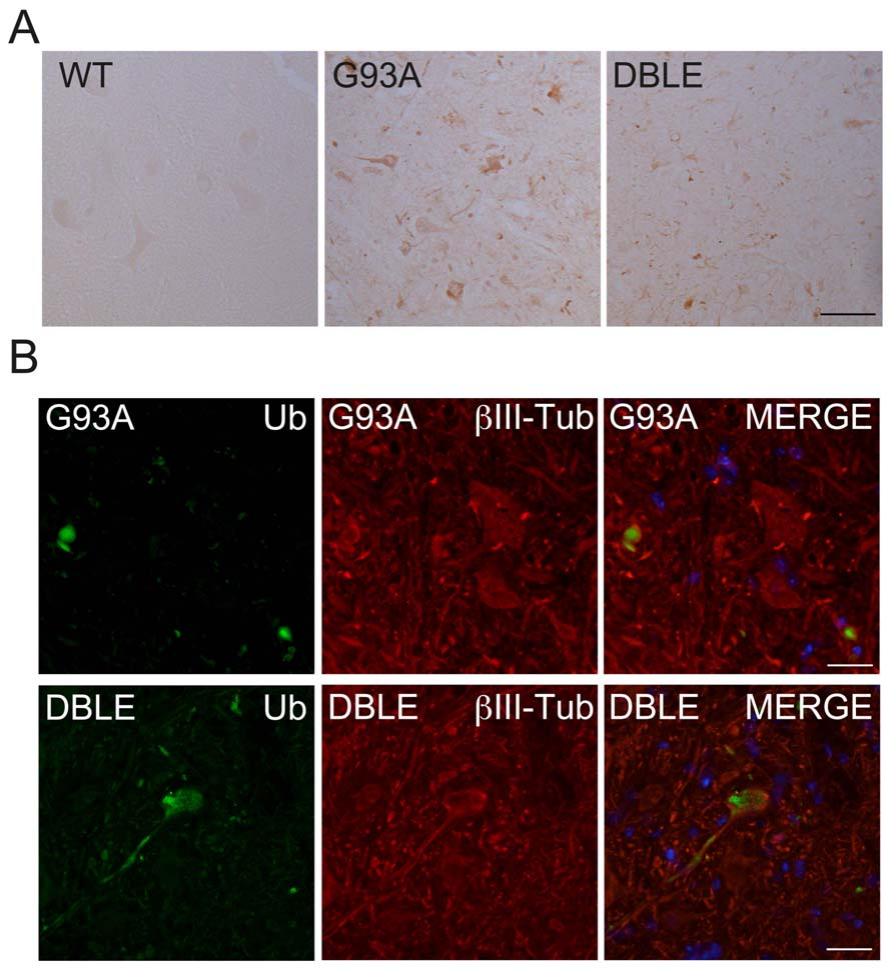
hHSJ1a alters ubiquitylation in SOD1^G93A^ spinal cord. (**A**) Immunohistochemistry with SEDI anti-SOD1 antibody on 120 day lumbar spinal cord from female mice showed no reactivity in WT tissue and punctate staining in G93A and DBLE. Scale bar 50 µm (**B**) Immunofluorescence of G93A or DBLE 90 day spinal cord from female mice, as indicated, showed enhanced ubiquitin staining (green) in motor neuron cell bodies and processes labelled with βIII tubulin antibody (red). Nuclei were stained with DAPI visible in MERGE panel. Scale bar 20 µm.

The localisation of SOD1 misfolding in tissue was investigated using an antibody to the SOD1 exposed dimer interface (SEDI) [36]. No staining was observed in the absence of SOD1^G93A^ overexpression, for example in the WT spinal cord. There was intense punctate staining in some motor neurons in the G93A spinal cords, as previously described [36]. Motor neurons were also immunoreactive for SEDI in the DBLE animals but the intensity of staining appeared to be reduced (Figure 7A), supporting the notion that there was less misfolded aggregated SOD1 present following hHSJ1a expression.

### hHSJ1a alters ubiquitylation in the spinal cord

HSJ1 expression has been shown to enhance protein ubiquitylation and HSJ1a can preferentially bind to ubiquitylated polyglutamine expanded huntingtin in mouse brain [25], [28], therefore, we tested the hypothesis that altered ubiquitylation could be mediating the reduction in SOD1 aggregation and enhanced motor neuron survival. Immunofluorescence of spinal cords with a pan-ubiquitin antibody revealed enhanced diffuse ubiquitin immunoreactivity in motor neuron cell bodies and processes compared to G93A spinal cord, where punctate ubiquitin immunoreactivity characteristic of inclusions was observed (Figure 7B). These data suggest that HSJ1a mediated a reduction in SOD1 aggregation in motor neurons late in the disease process through a mechanism potentially involving ubiquitylation and that this delays motor neuron cell death.

### HSJ1a reduces SOD1^G93A^ aggregation in a cell model

**Figure 8:**
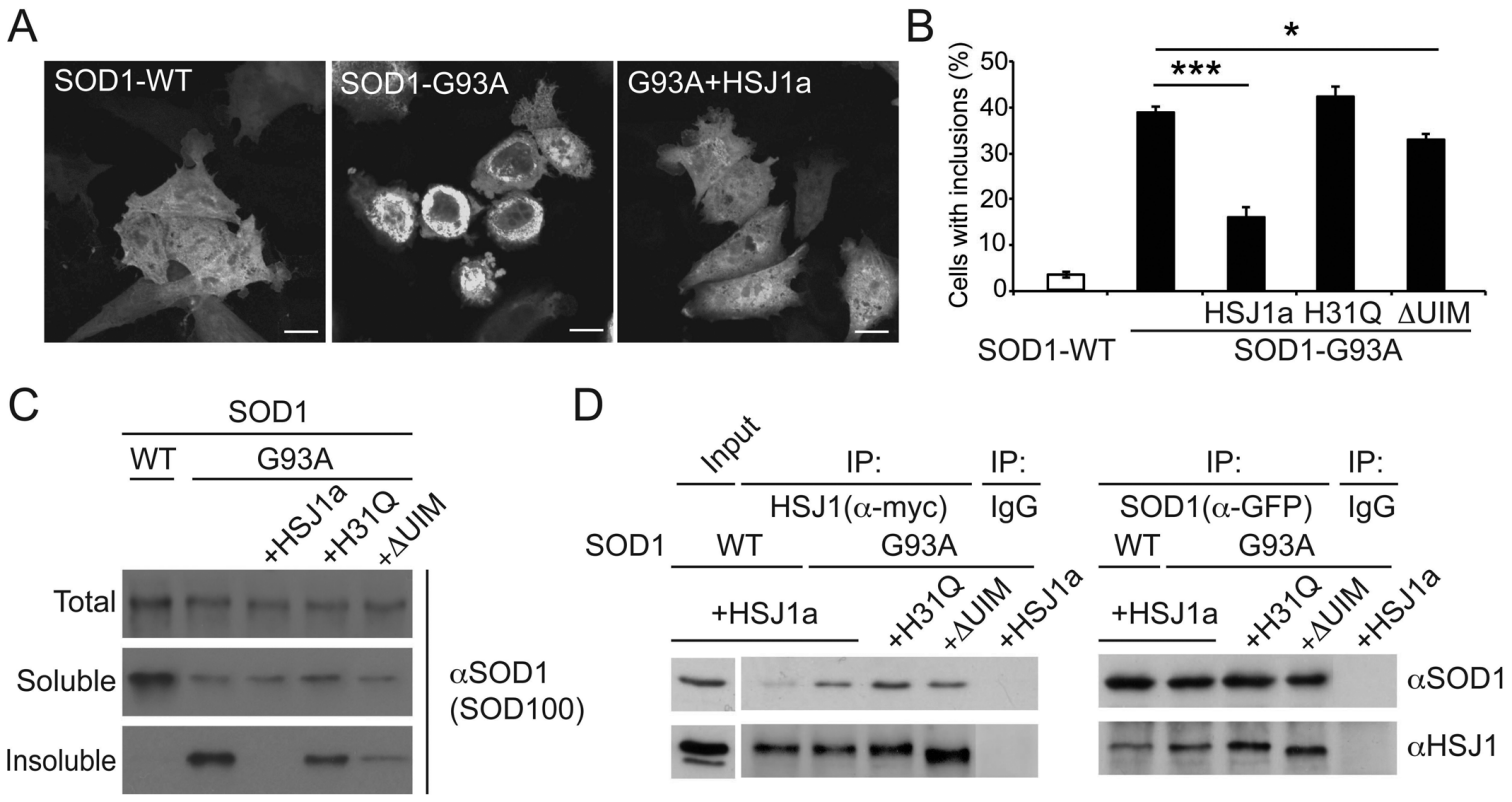
HSJ1a reduces inclusion formation in SK-N-SH cells. (**A**) Representative images of wild type (SOD1-WT) or SOD1^G93A^ mutant GFP-SOD1 (SOD1-G93A) localisation in transfected cells in the absence or presence of HSJ1a. Scale bars: 10 µm. (**B**) Incidence of inclusion positive cells determined by counting more than 400 cells per transfection condition in three independent experiments. Mean values ±SEM plotted for each group (*p<0.05, *** p<0.001). (**C**) Representative Western blots using anti-SOD1 (SOD-100) of SOD1 WT or G93A from total cell lysates (Total) compared to the soluble fraction (Soluble) or sedimented insoluble fraction (Insoluble) following differential sedimentation in the presence or absence of HSJ1a, HSJ1a J domain mutant (H31Q) or UIM mutant (ΔUIM). (**D**) Western blots with anti-SOD1 (SOD-100) or anti-HSJ1 (S653) showing reciprocal co-immunoprecipitation (IP) of SOD1 (anti-GFP) or anti-HSJ1a (anti-myc). HSJ1a had a higher recovery with SOD1-G93A compared to SOD1-WT. There was no co-precipitation with control non-specific antibody (IgG).

To probe the potential mechanisms and domain requirements of the hHSJ1a mediated reduction in SOD1 aggregation and improved motor neuron survival in the SOD1^G93A^ mouse model of ALS, we developed a cell model of SOD1^G93A^ aggregation based on the expression of GFP tagged SOD1 (GFP-SOD1) in SK-N-SH neuroblastoma cells, which do not express detectable endogenous HSJ1. Wild-type SOD1 (SOD1-WT) was evenly distributed throughout the cytoplasm and nucleus in over 95% of cells and was present in only the soluble protein fraction following sedimentation analyses (Figure 8A–C). In contrast, SOD1^G93A^ (SOD1-G93A) formed perinuclear inclusions in approximately 40% of cells and had a substantial insoluble fraction on sedimentation (Figure 8A–C). The expression of HSJ1a led to a significant reduction in SOD1-G93A inclusions and redistribution to an even cytoplasmic and nuclear staining pattern similar to the SOD1-WT. Furthermore, HSJ1a expression reduced the insoluble sedimentable component of SOD1-G93A. To test if the ability of HSJ1a to reduce SOD1-G93A aggregation was dependent on the J or UIM domains, we used a point mutation in the J domain that disrupts the interaction with Hsp70 (H31Q) or 4 point mutations that disrupt the function of the UIM domains (S219A/E222A/S262A/E265A) (ΔUIM) [25]. The J domain mutant H31Q lost the ability to suppress SOD1-G93A aggregation and inclusion formation, whereas the UIM mutant was partially functional, as it could still reduce aggregation and inclusion formation, but was not as efficient as wild-type HSJ1a. The interaction of HSJ1a with SOD1-WT and SOD1-G93A was compared by reciprocal co-immunoprecipitation (Figure 8D). HSJ1a bound preferentially to the mutant form of SOD1, although there was some detectable binding to SOD1-WT. The H31Q and ΔUIM mutants of HSJ1a retained the ability to bind SOD1-G93A. In fact, their binding was stronger than wild-type HSJ1a, suggesting that these mutants bind the misfolded client protein non-productively.

### HSJ1a enhances SOD1^G93A^ ubiquitylation

**Figure 9:**
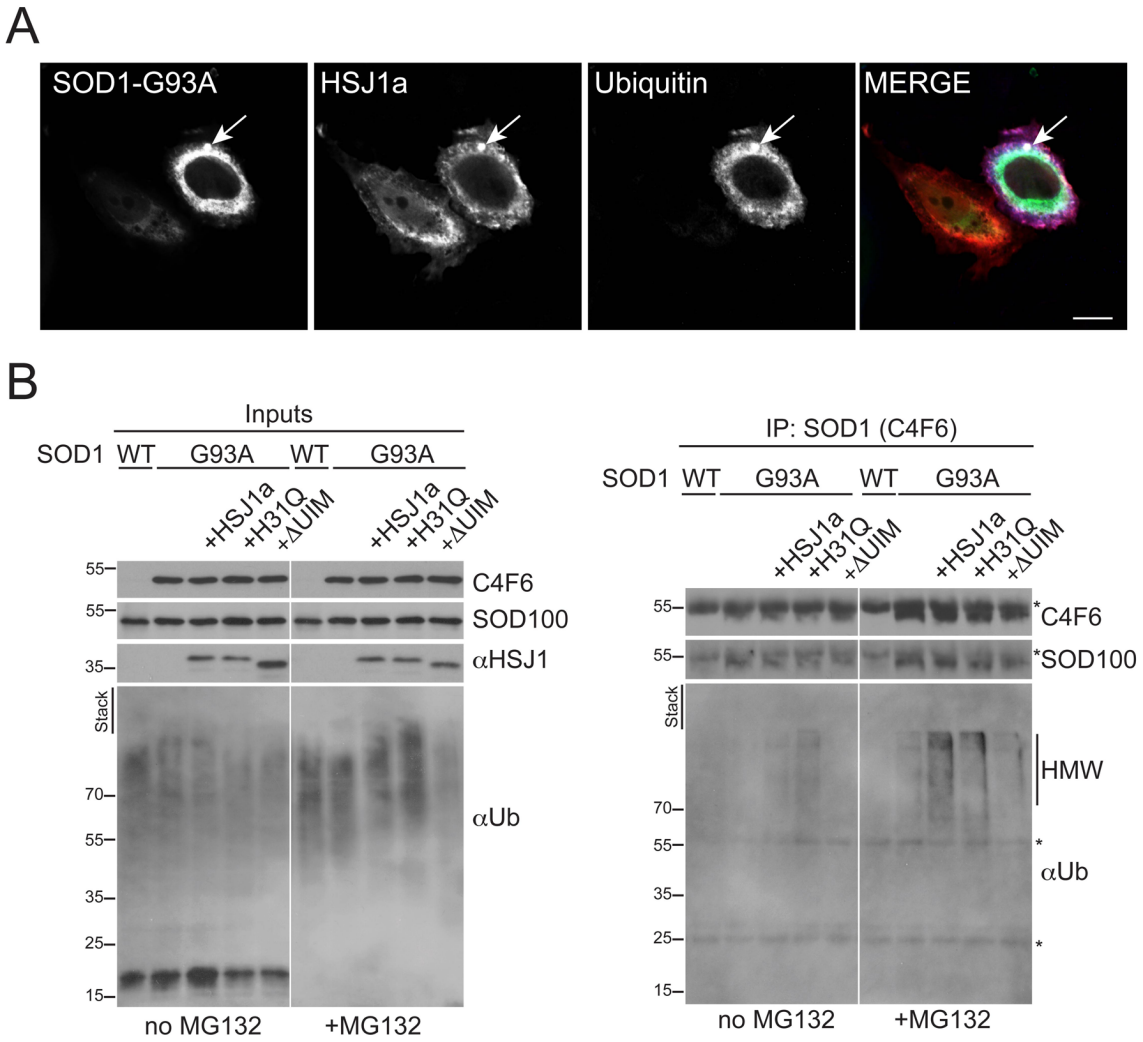
hHSJ1a enhances SOD1^G93A^ ubiquitylation. (**A**) Representative immunofluorescence image showing co-localization of mutant GFP-SOD1 (SOD1-G93A, green), myc-HSJ1a (S653, red) and myc-ubiquitin (pan Ub, blue) within an intracellular inclusion (arrowed) in SK-N-SH cells, following co-transfection and proteasome inhibition with MG132. Scale bar: 10 µm. (**B**) Western blots with anti-SOD1 (C4F6 or SOD100), anti-HSJ1 (S653) and pan-ubiquitin (Ub) antibody as indicated, showing input and C4F6-immunoprecipitated material from SK-N-SH cells transfected and treated with MG132 as indicated. An increase of ubiquitin reactivity of in the presence of WT HSJ1a was observed upon proteasome inhibition. The HSJ1a H31Q mutant did not stimulate proteasomal degradation of ubiquitylated SOD1, whereas the HSJ1a ΔUIM mutant did not stimulate SOD1-G93A ubiquitylation. Asterisks indicate IgG bands. The position of molecular weight markers is indicated on the left.

The potential of HSJ1a to alter SOD1 ubiquitylation was investigated further using the cell model. Triple label immunofluorescence revealed that both HSJ1a and ubiquitin were recruited to SOD1-G93A inclusions following proteasome inhibition with MG132 (Figure 9A). To test whether the mutant SOD1 was a target for HSJ1a mediated ubiquitylation, SOD1-G93A was immunoprecipitated with antibody C4F6 from cells co-transfected with HSJ1a, the J domain mutant H31Q, or the UIM mutant and blotted for ubiquitin (Figure 9B). The expression of HSJ1a or H31Q led to a small increase in HMW ubiquitin immunoreactivity in the cell lysate input, especially in the presence of proteasome inhibition as previously described [25]. In the absence of proteasome inhibition, the HMW ubiquitin immunoreactive smear in the C4F6 purified material was most pronounced in the cells transfected with the H31Q mutant. In contrast, proteasome inhibition revealed a strong increase in the C4F6 purified HMW ubiquitin immunoreactivity in the HSJ1a co-transfected cells (Figure 9B), suggesting that HSJ1a was enhancing mutant SOD1 ubiquitylation and stimulating its removal by the proteasome. The HSJ1a ΔUIM mutant did not enhance SOD1-G93A ubiquitylation, confirming that this effect was dependent on HSJ1a UIM domains.

## Discussion

ALS is recognized as a multifactorial disease with a range of pathways and cell types affected, leading to the demise of motor neurons and ultimately paralysis of muscles and death. The aggregation of mutant SOD1 has been implicated in the pathogenesis of fALS and the upregulation of molecular chaperones can protect against SOD1^G93A^ mediated motor neuron cell death [11], [12]. The upregulation of individual molecular chaperones, although effective in cell models, has been less effective in animal models [15], [17], [18]. This has led to the suggestion that upregulation of several chaperones is probably more effective than individual chaperones alone [37], and that protection may even require several chaperone machines. Here, we have shown that overexpression of HSJ1a can reduce SOD1 aggregation and improve motor neuron survival. This suggests that some molecular chaperones may be more effective than others at reducing protein aggregation and maintaining proteostasis in motor neurons.

HSJ1 is a member of a subfamily of DNAJB proteins that are particularly effective at preventing polyglutamine aggregation [38], but HSJ1 is unique in that it also contains two UIM domains that can bind ubiquitin [25]. HSJ1a overexpression reduced mutant huntingtin aggregation in mouse brain in a manner that was dependent on its J domain and UIM domains [28]. In the case of mutant SOD1, the J domain was also needed to reduce SOD1 aggregation in cells, suggesting a role for Hsp70. The J domain mutant could still bind to mutant SOD1 but the binding appears to be non-productive and suggests that HSJ1a co-operates with Hsp70 to actively prevent mutant SOD1 aggregation, rather than by a passive chaperone action. The involvement of the UIM domains of HSJ1a in protecting against mutant SOD1 was less clear, as the UIM mutant retained some partial activity, but was compromised relative to wild-type HSJ1a. We were not able to investigate the role of the J domain or UIM domain in the suppression of mutant SOD1 aggregation *in vivo* because of the relatively low recovery of mutant SOD1 by immunoaffinity purification techniques compared to mutant huntingtin [28]. In a cell model of mutant SOD1 aggregation, however, HSJ1a stimulated SOD1^G93A^ ubiquitylation and proteasomal degradation in a UIM dependent manner, suggesting that the altered ubiquitin immunoreactivity observed in the DBLE animals might be involved in the protective mechanism *in vivo*. Collectively, the data suggest that the combination of the different HSJ1a domains function together as a chaperone, cochaperone and pro-ubiquitylation factor to reduce mutant SOD1 aggregation. It is also possible that other members of this DNAJB subfamily of proteins that lack the UIM domain, but are effective against polyglutamine aggregation, may also be effective against mutant SOD1 in cells and *in vivo*.

In addition to the potential specialisation of certain chaperone anti-aggregation properties, it is likely that some chaperones are more important for motor neuron function and maintenance than others. In particular, mutations of some small heat proteins (HSPB1 and HSPB8) lead to dHMN and Charcot-Marie Tooth (CMT2) disease [39]. HSJ1 proteins are also important for motor neuron function or maintenance, as mutations in *DNAJB2* leads to motor neuropathy in man [30]. Whether the critical role of HSJ1 proteins is to prevent protein aggregation or deal with motor neuron specific client proteins still remains to be determined, but it is possible that a loss of HSJ1 chaperone activity could lead to enhanced aggregation of SOD1 or other proteins that could ultimately contribute to motor neuron cell death. This will need to be tested in animal models of HSJ1 loss of function to identify what the cellular consequences are of HSJ1 ablation for motor neuron biology. Similarly, overexpression of other dHMN or CMT2 chaperones may be beneficial for motor neuron survival. HSPB1 showed effects early, but not late, in disease [17], [20], and therefore might be complementary to HSJ1a overexpression. Such that a combination of the two chaperones could yield better protection. HSPB8 has yet to be tested in mouse models of fALS, although it is potentially a potent chaperone against fALS related proteins [19].

The overexpression of hHSJ1a improved motor neuron survival and muscle function in the SOD1^G93A^ mouse model, but did not prevent the SOD1^G93A^ mediated loss of body mass. Weight loss is a major determinant of the endpoint applied in our ethically approved protocols to reduce animal suffering, therefore, it is perhaps not surprising that we also did not observe any increase in lifespan in the DBLE mice. The lack of lifespan extension, or body mass retention, commensurate with the improved neuromuscular performance could reflect the relatively late effects we observed on SOD1 aggregation and motor neuron survival. Since HSJ1 is part of a larger chaperone complex, it is possible that increasing the expression of one chaperone from the complex cannot exert sufficient chaperone activity to fully correct protein misfolding in ALS. Thus, the reduction in SOD1 aggregation we observed may not be sufficient to protect motor neurons and neuromuscular function in the longer term and increase the lifespan of these mice. Reduced aggregation and increased motor neuron survival were only detectable close to end stage and the disease in this aggressive model may have been too advanced by this point to be delayed. It is unclear why the effect of hHSJ1a on SOD1 aggregation was only observed late in disease, as the level of hHSJ1a was relatively constant. This is unlike the case of mutant huntingtin and hHSJ1a line 52a where we observed a good spatiotemporal correlation between the level of hHSJ1a and reduction in mutant huntingtin aggregation [28]. It is possible that *in vivo* the mutant SOD1 is not fully accessible for HSJ1a chaperone activity until later in disease, either through a change in conformation or a post-translational modification, such as ubiquitylation. Alternatively, HSJ1a might only bind certain conformations of SOD1, similar to the way it prefers to bind high molecular weight complexes of mutant huntingtin [28], and that these species increase as the disease progresses. Interestingly, the proportion of mutant SOD1 that was insoluble increased between 90 and 120 days and this might reflect a change in the type of SOD1 aggregates or a failure of the cellular machinery to deal with the mutant protein later in disease. The effect of hHSJ1a on SOD1 is likely to be direct as hHSJ1a is present in a complex with SOD1 in DBLE spinal cord. Although both HSJ1a and HSJ1b have been reported to reduce SOD1^A4V^ inclusion formation in cells [30], mHSJ1b was not detected in the SOD1 complex suggesting that HSJ1a might be more efficient against mutant SOD1 *in vivo*. It was not possible to determine the composition of the HSJ1a: SOD1 complexes and whether they were monomeric, oligomers or aggregates. Nonetheless, the improved survival of motor neurons suggests that the SOD1 species targeted by HSJ1a are part of the pathological process and potentially important targets for chaperone based therapies.

In conclusion, our results show that over expression of HSJ1a ameliorates several disease signs at a late stage of disease progression in the SOD1^G93A^ mouse model of ALS. The late action of HSJ1a suggests that if methods could be developed for upregulating HSJ1a expression in motor neurons it could potentially be of benefit post-symptom onset. The data also confirm that reducing protein aggregation can lead to improvements in motor neuron survival and suggest that therapies aimed at improving protein quality control may enhance neuronal survival in ALS.

## Materials and Methods

### Ethical statement

All experiments were carried out under license from the UK Home Office in accordance with the Animals (Scientific Procedures) Act 1986 and following approval from the Institute of Neurology Ethical Review Committee. All surgery was performed under anaesthesia, efforts were made at all stages to minimize suffering, including the use of humane end points.

### Primary antibodies

HSJ1 antibody (S653) and anti-HSJ1a (16321) were generated as described [24] and affinity purified, SOD-100 was from Assay Designs (Ann Arbor, Michigan, USA), SEDI conformational antibody was a kind gift of Janice Roberston and Avi Chakrabartty (University of Toronto, Canada). C4F6 SOD1 antibody was a kind gift of Makoto Urushitani (Shiga University of Medical Science, Japan), GFP antibody was from Roche (Burgess Hill, West Sussex, UK), β-III-tubulin antibody was from Covance (Maidenhead, Berkshire, UK), pan-ubiquitin antibody was from Dako (Ely, Cambridgeshire, UK). c-Myc (clone 9E10) and β-tubulin antibody (clone TUB2.1) were from Sigma-Aldrich (Poole, Dorset, UK).

### Production of human HSJ1a transgenic mice

The hHSJ1a transgene PrP-hHSJ1a-poly(A) was created by cloning the bovine PrP promoter fragment [40] upstream of the hHSJ1a cDNA open reading frame [24] with a simian virus 40 poly(A) sequence. An 8 kb linearised, gel purified fragment in injection buffer (10 mM Tris-HCl pH 7.4, 0.25 mM EDTA) at 50 ng/µl was used for pronuclear injection of B6/CBA F1 blastocysts by Dr Jose Gonzalez (EMBL), with subsequent founder generation. Founders were screened by PCR using hHSJ1 specific primers F3 (5′-CAA TCA ATG GTG TCC CAG ATG ACC TGG-3′) and R3 (5′-CCA CAA CTA GAA TGC AGT G-3′). Positive founders were crossed to C57Bl/6 animals. The hHSJ1a line 61a was derived and hHSJ1a expression confirmed, then backcrossed onto a C57Bl/6×SJL background for use in this study.

### Mouse maintenance and breeding

All animals were maintained at the Denny Brown Laboratories, UCL Institute of Neurology, London, UK. Mice were housed at 21±1°C with relative humidity 55±10% and maintained on a 12-hour light/dark cycle with access to food (standard pellets) and water provided *ad libitum* via an overhead rack. At the onset of hindlimb paralysis, affected animals were provided with food pellets soaked in water at ground level to ensure sufficient nourishment and hydration. Transgenic mice expressing human SOD1^G93A^ mutant protein (TgN[SOD1^G93A^]1Gur; Jackson Laboratories, Bar Harbour) [31] were maintained by breeding male heterozygous carriers with female (C57BL/6×SJL) F1 hybrids. Crossing males heterozygous for SOD1^G93A^ mutant protein to females heterozygous for hHSJ1a transgene generated offspring of four genotypes used in this study. The presence of the SOD1^G93A^ mutation and hHSJ1a transgene was confirmed by PCR reaction from ear biopsies in all mice.

### Electrophysiological assessment of muscle force and motor unit number

Hind limb muscle force of female mice was assessed *in vivo* under general anaesthetic at 120 days of age. The animals were anaesthetised with 4.5% chloral hydrate (1 ml/100 g body weight) injected intraperitoneally (Sigma-Aldrich) and prepared for electrophysiological assessment of hind limb isometric muscle force. The distal tendons of the TA and EDL muscles in both hind limbs were exposed, dissected free from surrounding tissue and cut. The sciatic nerve was exposed in the mid-thigh region, and sectioned along with all of its branches except the deep peroneal nerve, which innervates the TA and EDL muscles. The hind limbs of the animal were rigidly secured to the table with stainless steel pins and the distal tendons of the TA and EDL attached to an isometric force transducer (Dyamometer UFI Devices, Welwyn Garden City, UK) via silk thread. The length of each muscle was adjusted to obtain maximal twitch tension. The muscles and nerves were kept moist with saline and experiments were carried out at approximately 22°C. Muscle contractions were elicited by stimulation of the sciatic nerve via platinum electrodes. Maximal tetanic contraction, was assessed by stimulating the sciatic nerves of both hind limbs with trains of stimuli at increasing frequencies of 40, 80 and 100 Hz for a total duration of 450 ms. The tetanic tension was measured with the aid of a computer and appropriate software (Picoscope, Pico Technology Limited). To determine the number of functional motor units in EDL muscle, stimuli of increasing intensity were applied to the sciatic nerve. This gradual increase in intensity resulted in stepwise increments in twitch tension due to successive recruitment of motor axons. The motor unit traces were recorded on a storage oscilloscope (Tektronix, Beaverton, OR, USA). The number of increments in twitch tension was counted to give an estimate of the number of motor axons present in the nerve.

### Histological assessment of motor neuron survival

At the end of the acute electrophysiological experiment, female animals were terminally anaesthetised with an overdose of chloral hydrate and perfused transcardially with 0.9% saline followed by 4% paraformaldehyde (PFA). The spinal cords were removed and the lumbar region (L2–L6) post-fixed overnight in PFA, and then cryoprotected in sucrose (30% in PBS) at 4°C. Serial transverse sections of spinal cord (20 µm) were cut on a cryostat, mounted onto poly-lysine coated slides and stained with gallocyanin, a Nissl stain. The sections were then dehydrated in increasing concentrations of alcohol, cleared in histoclear and then coverslips were mounted onto slides using DPX as a mounting solution. The number of motor neurons in the sciatic motor pool was assessed by counting the number of Nissl-stained motor neurons in every third spinal cord section in order to avoid counting the same cell twice in consecutive sections, using a light microscope (Leica, DMR). 40 spinal cord sections were assessed for each animal. Only large polygonal neurons with a distinguishable nucleus and nucleolus, visible at high magnification were included in the counts. This method of counting has previously been used to assess motor neuron survival [11], [33].

### Tissue preparation

At the end of the acute electrophysiological experiment, the TA and EDL muscles were removed from each hindlimb of male mice and weighed. In addition, in some animals, whole spinal cords were collected, snap-frozen in liquid nitrogen and stored at −70°C until being processed. They were then weighed, thawed by addition of PBS containing 5% protease inhibitor cocktail (Sigma-Aldrich) to a 1∶15 w/v ratio and homogenised by hand using an eppendorf-tube size pestle with subsequent sonication of the initial lysate until fully homogeneous. Lysates were then pre-cleared by brief centrifugation and used further for Western blotting, immunoprecipitation or differential sedimentation experiments.

### Assessment of bodyweight and lifespan

Male WT, hHSJ1a, G93A and DBLE mice were weighed at least twice weekly from 40 days of age until each G93A or DBLE mouse had lost 15% of their bodyweight, at which point they were humanely killed. After the last remaining G93A or DBLE mouse of each litter had lost 15% bodyweight all remaining mice of the litter were also humanely killed.

### Differential sedimentation

Pre-cleared cell or tissue lysate of 200 µl was subjected to first, slow centrifugation of 16,000 *g* at room temperature for 15 minutes resulting in supernatant-1 (S1, ‘soluble’) and pellet-1 (P1) fractions. An aliquot of S1 fraction was mixed with 4×SDS-PAGE sample buffer for future analysis while P1 was washed with 200 µl of lysis buffer and reconstituted in buffer containing 5% SDS by sonication with subsequent heating 98°C for 3 minutes. It was centrifuged at 225,000 *g* for 30 minutes at 4°C resulting in P2 and S2 fractions accordingly. The ‘insoluble’ P2 fraction was reconstituted in 2×SDS-PAGE sample buffer by sonication and boiling prior to analysis by Western blotting.

### SDS-PAGE, Western blotting and immunodetection

Protein lysates were mixed with 4×SDS-PAGE sample buffer containing (reducing conditions) or lacking (non-reducing conditions) 5% β-mercaptoethanol (Sigma-Aldrich) and subjected to SDS-PAGE. Proteins were then transferred to Protran nitrocellulose membranes by Western blotting using semi-dry transfer apparatus (Bio-Rad). Nitrocellulose membranes were blocked with 5% non-fat dried milk with 0.1% (v/v) Tween-20 in PBS for 1 h at RT or at 4°C overnight. Membranes were then incubated with appropriate primary (S653 1∶3000; SOD-100 1∶2000; C4F6 - 1∶3000; pan-Ub 1∶1000; TUB2.1 1∶2000) and secondary HRP-conjugated antibodies (Perbio Science UK, Cramlington, Northumberland, UK) and signal was detected using ECLplus reagents (GE Healthcare, Chalfont St Giles, Buckinghamshire, UK) and X-ray film (Fuji Medical UK, Bedford, Bedfordshire, UK). Films were processed using a Konica-Minolta developer.

### Densitometry

Densitometry of Western blots was performed using an EPSON Perfection V350 photo scanner with manufacturer's software. Developed films were scanned and the average pixel density (OD) for each band was measured using ImageJ software. The OD of an area devoid of bands was subtracted from the values obtained for bands of interest in order to normalize OD against background. Relative expression was determined by dividing the normalized OD of bands of interest by the OD of mouse HSJ1a bands or as a percentage of β-tubulin bands intensity as specified for each experiment.

### Immunoprecipitation (IP) and co-IP

400 µl of protein lysate was incubated with 25 µl of protein-G Dynabeads (Invitrogen) and capture antibody (S653 2 µl; C4F6 1 µl; anti-GFP 1 µl; 9E10 1 µl) for 1 h at room temperature or overnight at 4°C on a rotating wheel. Precipitated material was eluted from beads by heating for 3 min at 98°C in 30 µl of 4×SDS-PAGE sample buffer and subjected to SDS-PAGE and Western blotting with antibodies against pulled-out protein (IP) or proteins it has formed a complex with (co-IP).

### Immunostaining

For immunostaining, 10 µm serial lumbar spinal cord sections were cut on a cryostat. Sections were briefly incubated in PBS 0.1% Triton X-100 plus 3% H_2_O_2_ and then blocked in PBS containing 0.1% Triton x-100 and 5% serum, before exposure to primary antibody diluted in blocking solution ((16321 1∶100; SOD-100 1∶200; SEDI 1∶500) and appropriate secondary antibody (1∶100 v/v with block) and then developed using diamino benzidine (DAB). For immunofluorescence, spinal cord cryosections were rehydrated in PBS and permeabilised by incubation in PBS with 0.5% Triton-X100 (v/v) for 10 min. After being washed twice for 10 min in PBS, sections were blocked for 40 min at room temperature in blocking solution (10% FCS (w/v), 0.1% Triton-X100 (v/v), 3% normal donkey serum (Sigma-Aldrich) in DMEM/F12). Primary antibody (SEDI 1∶100; β-III-tubulin 1∶500; pan-Ub 1∶200) was diluted in blocking solution and incubated with sections at 4°C overnight. Sections were then washed twice in PBS for 15 min and incubated for 1 h at room temperature in fluorophore-conjugated donkey secondary antibody from Jackson ImmunoResearch (Stratech, Newmarket, Suffolk, UK) in blocking solution. After washing twice with PBS, sections were stained with DAPI (Sigma-Aldrich) and mounted using fluorescent mounting media (Sigma-Aldrich) and cover slides (VWR). Images were captured using a Zeiss LSM700 confocal microscope with ZEN software.

### Cell culture, plasmids and transfection

To produce GFP-tagged SOD1 (GFP-SOD1), SOD1-WT and SOD1-G93A were amplified from pTRE-SOD1-WT-CFP and pTRE-SOD1-G93A-CFP (gift from Rick Morimoto, Northwestern University, USA) using SOD1 specific primers engineered to introduce restriction endonuclease sites (Eco RI and Bam HI). DNA was initially ligated into a pGEM-T vector (Promega, Southampton, Hampshire, UK) before ligation into the pEGFP C1 vector (Clontech, Mountain View, California, USA). Human neuroblastoma SK-N-SH cells (ECACC, Porton Down, UK; catalogue number 86012802) were maintained at 37°C and 5% CO_2_ for 24 hrs in DMEM/F12 containing 10% FCS from Gibco, (Life Technologies Ltd, Paisley, Renfrewshire, UK) and 50 µg/ml gentamicin (Gibco) to reach 70% confluency in 6 well plates (BD Falcon, Oxford, Oxfordshire, UK) or 8 well chamber slides (VWR, Lutterworth, Leicestershire, UK). Cells were then transfected with 1 µg/well of total DNA which was a 1∶1 mixture of pEGFP-SOD1 (WT or G93A mutant) and pCMV-Tag3a-HSJ1a (wild type, H31Q or UIM mutants described previously [24], [25]) or empty vector using Lipofectamine and Plus reagents from Invitrogen (Life Technologies Ltd, Paisley, Renfrewshire, UK) according to manufacturer's instructions. For SOD1 ubiquitylation experiments, the plasmid DNA ratio was 2∶1∶2 of pEGFP-SOD1, pCMV-Tag3a-HSJ1a and pCW7-myc-Ub respectively. Cells were treated with 50 µM MG132 proteasome inhibitor (Enzo Life Sciences, Plymouth, UK) or DMSO vehicle for 4 hours prior to lysis. 24 hrs post transfection cells were lysed in 200 µl/well of RIPA buffer containing 5% of protease inhibitor cocktail (Sigma-Aldrich), sonicated 3×5 seconds on ice, cleared, and used for immuno-precipitation or differential sedimentation. Cells in chamber slides were fixed with 4% paraformaldehyde and used for inclusion counts on Nikon Eclipse 80i epi-fluorescence microscope (Nikon UK Ltd., Kingston Upon Thames, Surrey, UK). Images were taken using a Zeiss LSM 700 confocal microscope (Carl Zeiss UK, Cambridge, Cambridgeshire, UK).

### Statistical analysis

Statistical significances were calculated using ANOVA incorporating a Student Newman Kreuls multiple comparisons test. Significance was set at p<0.05.
